# Supplementary figures and images for: Post-Transcriptional Regulation of Alpha One Antitrypsin by a Proteasome Inhibitor
Source: Int J Mol Sci. 2020 Jun 17;21(12):4318. doi: 10.3390/ijms21124318 (PMC7352753; doi:10.3390/ijms21124318)

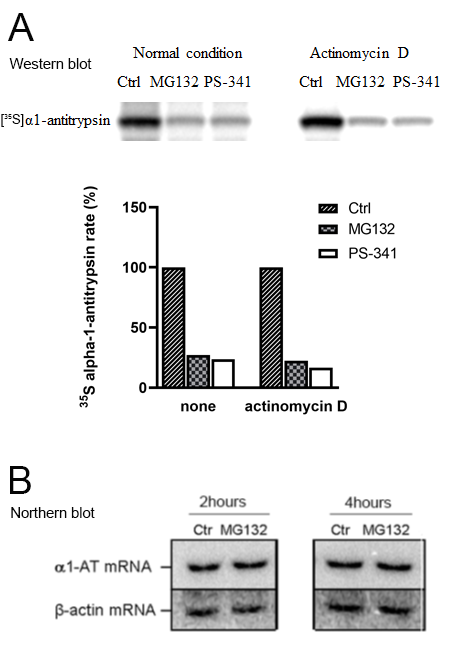

Supplement: Supplementary file 1 [file ijms-21-04318-s001.zip › FigureS2 IJMS new.tif]

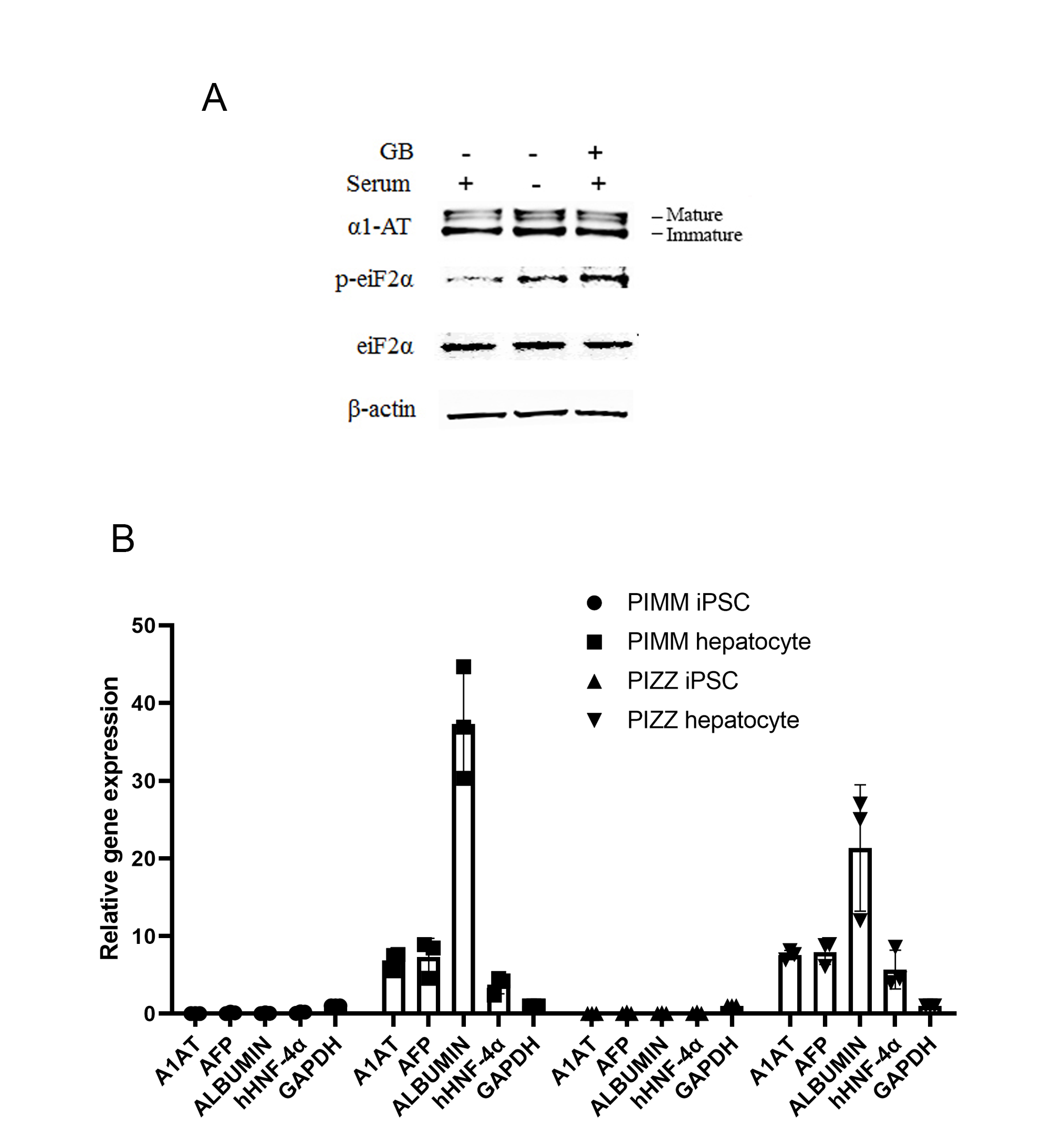

Supplement: Supplementary file 1 [file ijms-21-04318-s001.zip › FigureS3 IJMS1.tif]

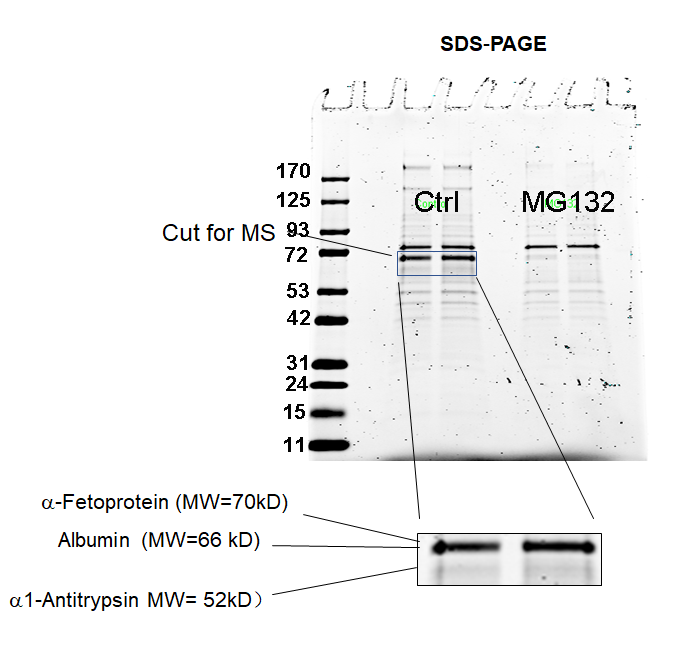

Supplement: Supplementary file 1 [file ijms-21-04318-s001.zip › FigureS4 IJMS new.tif]

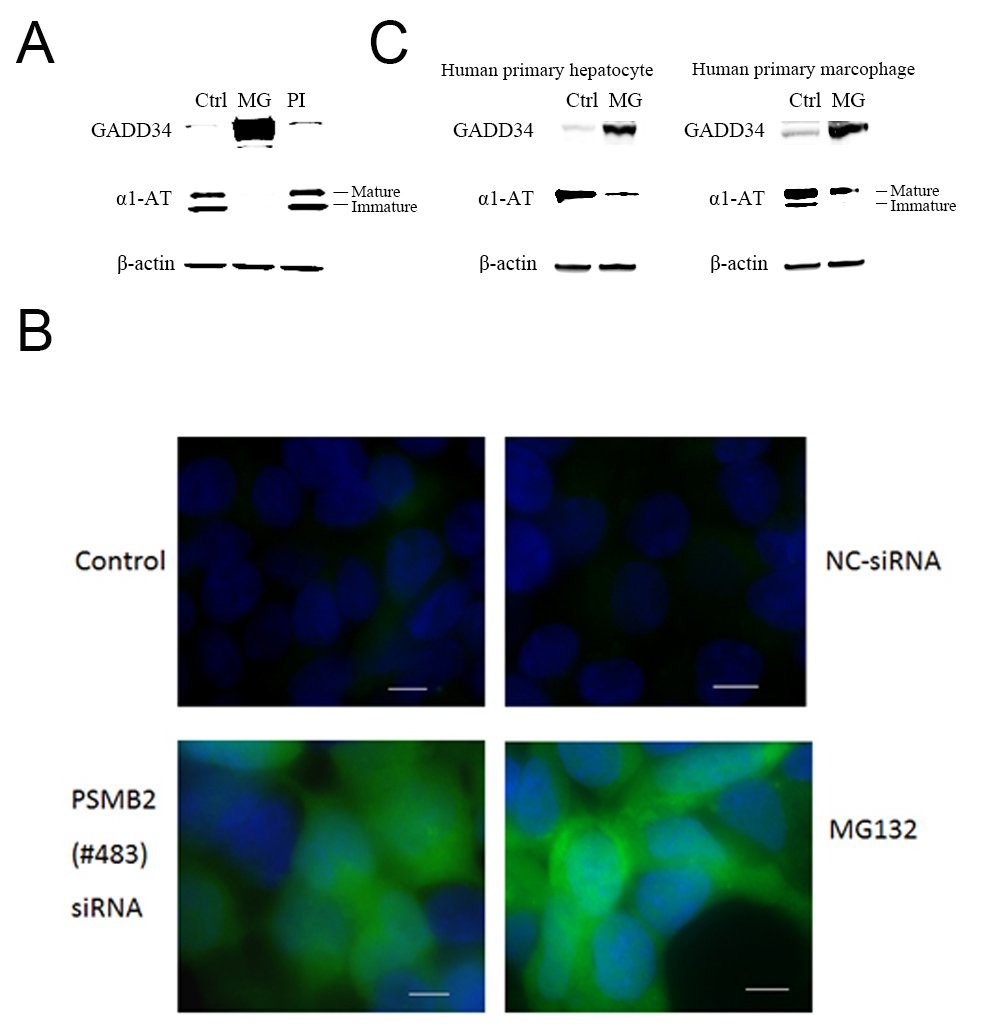

Supplement: Supplementary file 1 [file ijms-21-04318-s001.zip › FigureS1 IJMS.tif]
